# Polyethylene Glycol (PEG)-Based Wet-Adhesive Absorbable Bone Wax for Osseous Hemostasis and Repair

**DOI:** 10.3390/ijms27010276

**Published:** 2025-12-26

**Authors:** Huiqiang Cheng, Aiping Yang, Guoyu Lv, Heng Zheng, Hong Li

**Affiliations:** College of Physics, Sichuan University, Chengdu 610065, China; chq19831150689@outlook.com (H.C.); yangaiping@scu.edu.cn (A.Y.); lgy929@126.com (G.L.)

**Keywords:** absorbable bone wax, PEG, wet adhesion, hemostasis

## Abstract

The non-absorbability and biological inertia of traditional bone wax often result in foreign body retention, inhibit bone healing, and increase the risk of infection. Herein, a novel absorbable bone wax was developed by mixing polyethylene glycol (PEG), tannic acid (TA), sodium carboxymethyl cellulose (CMC), and low-molecular-weight poly-L-lactic acid (PLLA) via one-pot method. Among them, PEG functions as the matrix, endowing it with plasticity and hydrophilicity, thereby facilitating its facile, close fit to the surface of bone defects. TA, with its profusion of phenolic hydroxyl groups, bestows the material with exceptional adhesion properties. The hydroxyl and carboxyl groups in the CMC molecular structure are capable of forming hydrogen bond networks with PEG and TA. Concurrently, hydrophobic PLLA reinforces the hydrogen bond network structure. Absorbable bone wax maintains excellent adhesion in water. The obtained wax shows an adhesion strength of 27.07 kPa and can maintain adhesion to pig bone for 6.0 h in an aqueous environment. In particular, after being soaked in water for 24 h, the bone wax only dissolves on the surface, while the core remains intact. The results of the porcine sternum implantation experiment showed that the hemostatic performance of this bone wax was comparable to that of non-absorbable bone wax. Furthermore, the absorbable bone wax was gradually absorbed, leading to better bone tissue healing. This absorbable formulation could eliminate the 5–15% infection rate associated with traditional bone wax.

## 1. Introduction

Bones are known to contain a rich network of blood vessels and bone marrow [1,2]. In the event of damage to the vascular system due to external trauma or surgical incisions, bleeding at the bone wound site may occur. It is imperative that hemostasis is achieved on time to avert the occurrence of subsequent pathological consequences, such as tissue necrosis. Failure to do so may potentially compromise the patient’s life safety [3]. Traditional bone wax, consisting primarily of beeswax and softeners, functions as a mechanical barrier to seal bleeding sites of bone wounds (e.g., sternotomy, iliac bone harvesting, pituitary tumor resection, etc.) to achieve hemostasis. Generally, it exhibits excellent plasticity and a smooth texture, facilitating facile adhesion to bone surfaces [4,5]. Additionally, it is cost-effective and readily available, thus being widely applied in orthopedic, neurosurgical, and cardiothoracic surgeries, as well as in dental and maxillofacial surgeries and surgical tool modifications for auxiliary hemostasis [6]. However, traditional bone wax, due to its inherent inertness and non-biodegradability, also shows some disadvantages and induces severe adverse effects, such as foreign body reactions, bone healing delay, and infections after implantation in the human body [7,8,9,10,11,12,13].

Given the aforementioned limitations in the performance of traditional bone wax materials, absorbable bone wax has emerged as a novel strategy. The absorbable bone wax is mainly composed of synthetic polymers, natural polymers, inorganic materials, and their composite materials. Synthetic polymers include alkylene oxide copolymers such as Ostene [14], polyethylene glycol (PEG) [15], and cyanoacrylates. Ostene and PEG are highly water-soluble, resulting in suboptimal hemostatic efficacy [16,17,18], while cyanoacrylates exhibit a lack of plasticity and flexibility [19]. Examples of natural polymers include gelatin foam [20], oxidized cellulose [21,22], microfibrillar collagen [20], chitosan [23,24,25,26,27], etc. These materials have been demonstrated to exert their hemostatic effect by means of compressing the bleeding area. However, they are not generally considered suitable for use in cases of irregular bone defects, a common occurrence in cases of bone fractures [23,26]. Inorganic materials (e.g., calcium phosphate, calcium sulfate, hydroxyapatite, and bioactive glass) have been demonstrated to exhibit favorable biocompatibility and osteoconductive properties [15,28,29]. However, their inherent fragility, limited capacity for plasticity, and the challenge of regulating degradation rates constrain their potential for bone wax application.

Polyethylene glycol (PEG) is widely used in the biomedical field owing to its favorable absorbability, biocompatibility, and low toxicity [15]. It has been approved by the FDA for use in diverse fields, such as surface modification, bio-coupling, drug delivery, and tissue engineering [30,31,32]. However, polyethylene glycol (PEG), when utilized as bone wax, exhibits notable limitations: first, its excessively rapid dissolution results in the failure of hemostasis [33,34]; second, its inadequate adhesion to osseous tissue leads to detachment during surgical wound irrigation [18,35]. Hence, adjusting the adhesion and solubility of PEG in water is a crucial step for successfully preparing PEG-based absorbable bone wax. Drawing inspiration from the structure of mussel protein [36,37], Tannic acid (TA) and various catechol-containing compounds (such as dopamine, tea polyphenols [38], and lignin [39]) have been used to improve adhesion. However, tannic acid’s hydrophilic nature may reduce its adhesion in environments with large amounts of body fluids, particularly in wounds with significant bleeding. Therefore, it needs to be modified to enhance its water resistance. The molecular structure of CMC contains a large number of hydroxyl and carboxyl groups [40], which facilitates the formation of intermolecular hydrogen bonds and forms hydrogen bonds with polar groups on PEG [31,41] and TA [32,42,43]. This thereby improves the water resistance of the system. CMC’s hydroxyl/carboxyl groups form hydrogen bond networks with both TA and PEG, creating a crosslinked structure that resists water penetration. Simultaneously, hydrophobic PLLA domains create water-resistant microregions that maintain adhesion even when blood-soaked. We hypothesized that combining PEG’s biocompatibility with TA’s adhesive phenolic groups, CMC’s hydrogen-bonding capacity, and PLLA’s hydrophobic reinforcement would create a synergistic system maintaining adhesion in blood-soaked environments—a critical unmet need.

In summary, this study designed an absorbable polyethylene glycol (PEG)-based bone wax, leveraging its excellent plasticity, absorbability, and biocompatibility. Meanwhile, tannic acid (TA) and carboxymethylcellulose (CMC) were incorporated into the PEG-based bone wax. TA and CMC can form intermolecular hydrogen bonds with PEG to construct a polymer network structure, which effectively enhances the adhesion of the bone wax to osseous tissue and achieves better occlusion of bleeding sites. Furthermore, low-molecular-weight poly-L-lactic acid (PLLA) was introduced into the aforementioned molecular structure to make the polymer network more compact, while hydrophobic PLLA can further increase the adhesion of absorbable bone wax in water. Meanwhile, the absorbable PEG-based bone wax was successfully fabricated via a one-pot method by mixing PEG, TA, CMC, and low-molecular-weight PLLA under mild conditions. Subsequently, a series of material property characterizations was systematically conducted to evaluate its water solubility, adhesive strength, and sealing property in water as well. Moreover, FTIR, DSC, SEM, and rheological curve tests were carried out. To comprehensively assess its clinical applicability, in vivo hemostatic evaluations were conducted on a pig sternum defect hemorrhage model, followed by histological analysis at predetermined time points to evaluate both the immediate hemostatic efficacy and the long-term osseous tissue healing process, including neovascularization, fibrous tissue, and new bone formation.

## 2. Results and Discussion

### 2.1. FTIR, DSC, SEM, and Rheology

**Figure 1:**
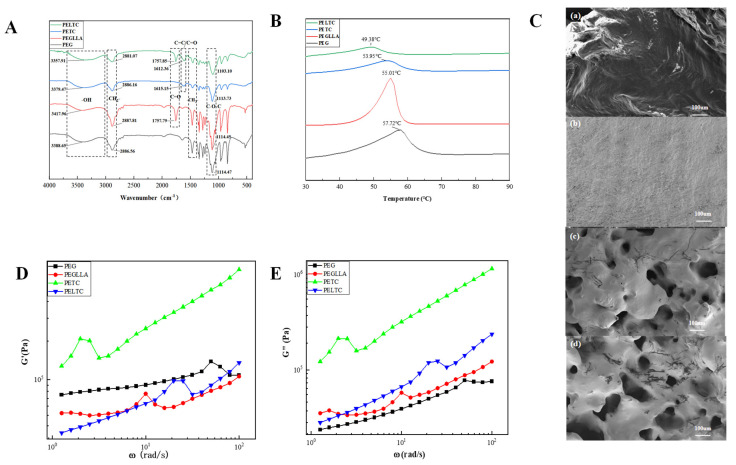
(**A**) FTIR spectrum of absorbable bone wax. (**B**) DSC curve of absorbable bone wax. (**C**) SEM of absorbable bone wax ((**a**–**d**) represent PEG, PEGLLA, PETC, and PELTC, respectively). (**D**,**E**) Rheological curves of absorbable bone wax.

The strength of intermolecular hydrogen bonds can be characterized by FTIR. Figure 1A shows the infrared spectrum of absorbable bone wax, which can characterize the intermolecular forces. As shown in Figure 1A, the wide bands at 3350–3650 cm^−1^ and 2700–2975 cm^−1^ were owing to the stretching vibration of O–H and C–H bonds, respectively. The bands at 1720–1770 and 1050–1150 cm^−1^ were owing to the stretching vibration of C=O and C–O–C bonds. The band at 1350–1490 was due to the in-plane bending vibration of C–H bonds. The hydroxyl stretching vibration peaks of PEG and PLLA are 3388.65 cm^−1^ and 3417.96 cm^−1^, respectively. The hydroxyl stretching vibration peaks of PEG, PETC, and PELTC are 3388.65 cm^−1^, 3379.47 cm^−1^, and 3357.91 cm^−1^, respectively. The infrared characteristic peaks of PETC and PELTC undergo a red shift, and the peak shapes of PETC and PELTC become broader. It is explained that hydrogen bonds exist in both PETC and PELTC and that the hydrogen bond forces in PELTC are stronger [44,45,46]. Hydrogen bonds cause a redistribution of the electron density in the O–H bond, weakening the bond energy of the O–H bond and reducing the corresponding vibration frequency, resulting in a red shift in the infrared characteristic peak of the hydroxyl group.

Absorbable bone wax must demonstrate a certain degree of heat resistance to ensure that it does not melt when implanted in the human body. At the same time, the addition of PLLA, TA, and CMC will also interfere with the crystallization properties of PEG. The DSC analysis is employed to assess the heat resistance and crystallization properties of the absorbable bone wax. Figure 1B shows the DSC curve of absorbable bone wax. The melting points of PEG, PEGLLA, PETC, and PELTC are all greater than 37 °C, and their heat resistance meets requirements, but their crystallization properties differ. PEG has the highest melting point and largest enthalpy of fusion, at 57.72 °C and 189.4 J/g, respectively, indicating that it has the optimal crystallization properties. The melting point and melting enthalpy of PEGLLA are 55.01 °C and 180.9 J/g, respectively, both of which are slightly lower than those of PEG. This indicates that the introduction of PLLA disrupts the ordered arrangement of the molecular chains, resulting in reduced crystallization ability of PEGLLA [47,48]. The melting point and melting enthalpy of PETC are 53.95 °C and 80.32 J/g, respectively. The main reason for the low melting point and melting enthalpy of PETC is the formation of intermolecular hydrogen bonds, which disrupt the regular arrangement of the molecular chains and increase crystal defects [49]. In addition, the irregular structures in CMC and TA molecules also affect crystallization performance. PELTC has the lowest melting point and smallest enthalpy of fusion, at 49.38 °C and 46.51 J/g, respectively. This is due to the combined effects of intermolecular hydrogen bonding and rigid structures such as PLLA, CMC, and TA, which interfere with the orderly arrangement of molecular chains, resulting in the poorest crystallization performance. PELTC’s reduced crystallinity (melting enthalpy 46.51 vs. 189.4 J/g for PEG) enables better conformability to irregular bone surfaces while maintaining >37 °C melting point for in vivo stability.

Hydrogen bonds between molecules not only affect the crystallization properties of absorbable bone wax but also affect its microstructure. The SEM of the absorbable bone wax is illustrated in Figure 1C. The PEG sample exhibited a smooth cross-section, devoid of any distinctive microscopic structure. The PEGLLA sample displayed a dense, flocculent structure, characterized by the presence of small pores between the molecular chains. This phenomenon may be attributed to the incorporation of PLLA, which disrupts the ordered arrangement of the molecular chains, resulting in the formation of pores during the stacking process and consequently affecting its crystallization process. It has been demonstrated that both PETC and PELTC exhibit a unique porous network structure. The pore sizes of PETC and PELTC are approximately 50 um. This phenomenon is attributed to the formation of intermolecular hydrogen bonds, which significantly weaken the crystallization ability of PEG. As a result, the molecular chains are unable to stack in an orderly manner. Concurrently, the interaction of intermolecular hydrogen bonds connects the molecular chains, ultimately forming a porous network structure [50]. The reason why absorbable bone wax has good adhesion in a humid environment may be due to hydrogen bonding contributing to the formation of a porous network structure. The specific reasons will be discussed in Section 2.4.

Adhesion is the process by which absorbable bone wax interacts with bone tissue at the molecular level to form bonds. It involves stages such as interface contact and molecular chain diffusion. The storage modulus and loss modulus regulate adhesion performance by influencing these stages [51]. The storage modulus (G’) reflects the energy stored in elastic deformation under periodic external forces, which depends on the “elastic network strength” formed by intermolecular forces, chain entanglement, or crosslinking. The loss modulus (G”) reflects the energy dissipated by viscous deformation (chain segment sliding, internal friction) of the material and is directly related to the “difficulty” of molecular chain movement and the “magnitude of internal friction” [52,53]. The rheological curves of absorbable bone wax are shown in Figure 1D,E. PEG has a moderate storage modulus (G’), but its loss modulus (G”) is the lowest. This is because PEG is a crystalline polymer, and the crystalline regions form physical crosslinking points that effectively restrict the large-scale sliding of molecular chains, significantly improving elasticity. Therefore, its G’ is moderate. The low G” of PEG is due to its high molecular chain flexibility and low internal friction. Compared to PEG, PEGLLA has a lower G’ but a higher G”. This is because the addition of PLLA reduces the crystallinity of PEG, thereby hindering the formation of a physical crosslinking network. Furthermore, PLLA segments contain rigid methyl (-CH3) and ester (-COO-) groups, which are significantly less flexible than PEG’s ether bond segments. This results in increased internal friction during PEG segment rotation and slippage. The storage modulus of PETC is the highest, which is attributed to the synergistic effect of the physical crosslinking network formed by PEG crystallization and physical crosslinking network constructed by hydrogen bonds. Furthermore, PETC exhibits the highest loss modulus, which can be attributed to the presence of a substantial number of rigid groups within TA and CMC in PETC. This characteristic impedes molecular chain flexibility and leads to increased internal friction. It has been demonstrated that both G’ and G” of PELTC are lower than those of PETC. This is primarily because the addition of PLLA disrupts PEG crystallization, leading to damage to the physical crosslinking network. Although PLLA has a reinforcing effect on the hydrogen bond network, the enhancement of the modulus by the hydrogen bond crosslinking network is far smaller than the loss of the modulus caused by the structural damage of the crystalline physical crosslinking network. Therefore, the G’ of PELTC is lower than that of PETC. The lower G’’ value of PELTC compared to PETC is due to the lower molecular weight of PLLA, which has shorter chains and extremely low entanglement, resulting in good intrinsic fluidity. This is equivalent to introducing a “molecular-level lubricant” into the system. Therefore, the internal friction of PELTC is lower than that of PETC. Adhesion strength comparison: PEG < PEGLLA < PETC < PELTC. The primary reason is that PEG exhibits the lowest G” value, making it difficult to deform and resulting in insufficient interfacial contact, thus yielding the poorest adhesion. PEGLLA exhibits a lower G’ but higher G”, which promotes full interfacial contact and consequently enhances adhesion. PETC exhibits the highest values for both G’ and G”, enabling full interface contact and thus superior adhesion. However, excessively high G’ tends to cause stress concentration, resulting in adhesion slightly inferior to that of PELTC. PELTC exhibits moderate G’ and relatively high G”, which maintains sufficient interface contact while preventing stress concentration caused by excessively high G’. Consequently, PELTC offers the best adhesion properties.

### 2.2. Plasticity

**Figure 2:**
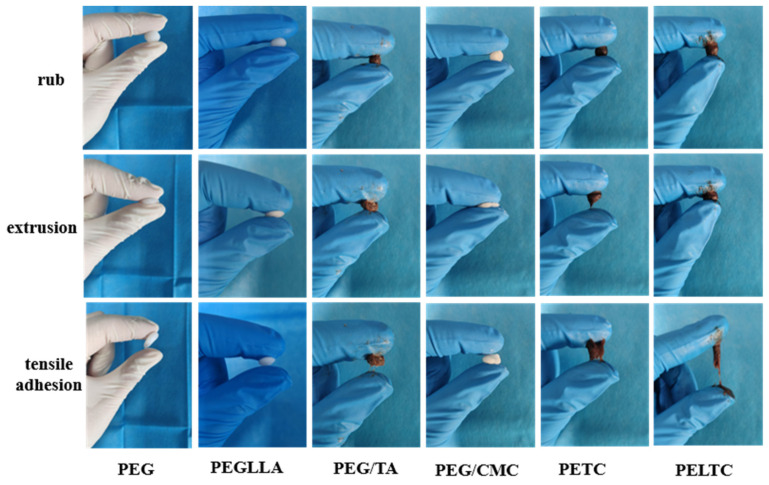
The plasticity and tensile adhesion of absorbable bone wax.

Absorbable bone wax should have the same plasticity as traditional bone wax and be easy to shape to allow for easier manipulation during surgery. By repeatedly kneading, squeezing, and stretching the absorbable bone wax with the fingers, one can preliminarily assess its plasticity and tensile adhesion. The plasticity test of traditional bone wax can be found in the Supporting Materials. As shown in Figure 2, all six samples could be kneaded into balls and withstand compression, demonstrating good plasticity and ease of use in clinical settings. This is because both PEG400 and low-molecular-weight PLLA are liquid, have short molecular chains, have few interchain entanglements, and have strong fluidity, enabling shaping through intermolecular sliding. The addition of PEG400 or low-molecular-weight PLLA acts as a plasticizer for the absorbable bone wax, so all six samples exhibit good plasticity. At the same time, in order to achieve rapid hemostasis at the site of bone defects, absorbable bone wax must also have a certain degree of tensile adhesion. The samples exhibited significant differences in tensile adhesion. PEG, PEGLLA, and PEG/CMC exhibited poor adhesion; finger separation and sample peeling occurred almost simultaneously. PEG/TA, PETC, and PELTC exhibit excellent adhesion properties. This indicates that the addition of TA significantly improves tensile adhesion. The formation, deformation, and crack propagation of the fiber structure led to significant mechanical dissipation, thereby greatly enhancing the adhesive strength [44]. Adhesion strength can be preliminarily assessed based on the adhesion points and the length of fiber elongation. The greater the number of adhesion points and the longer the length of fiber elongation, the better the material’s adhesion strength. PEG/TA, PETC, and PELTC all exhibit adhesion points, but their fiber elongation lengths differ. The approximate fiber elongation lengths for the three materials are 0.5 cm, 1.0 cm, and 2.0 cm, respectively.

### 2.3. Dissolution Performance

**Figure 3:**
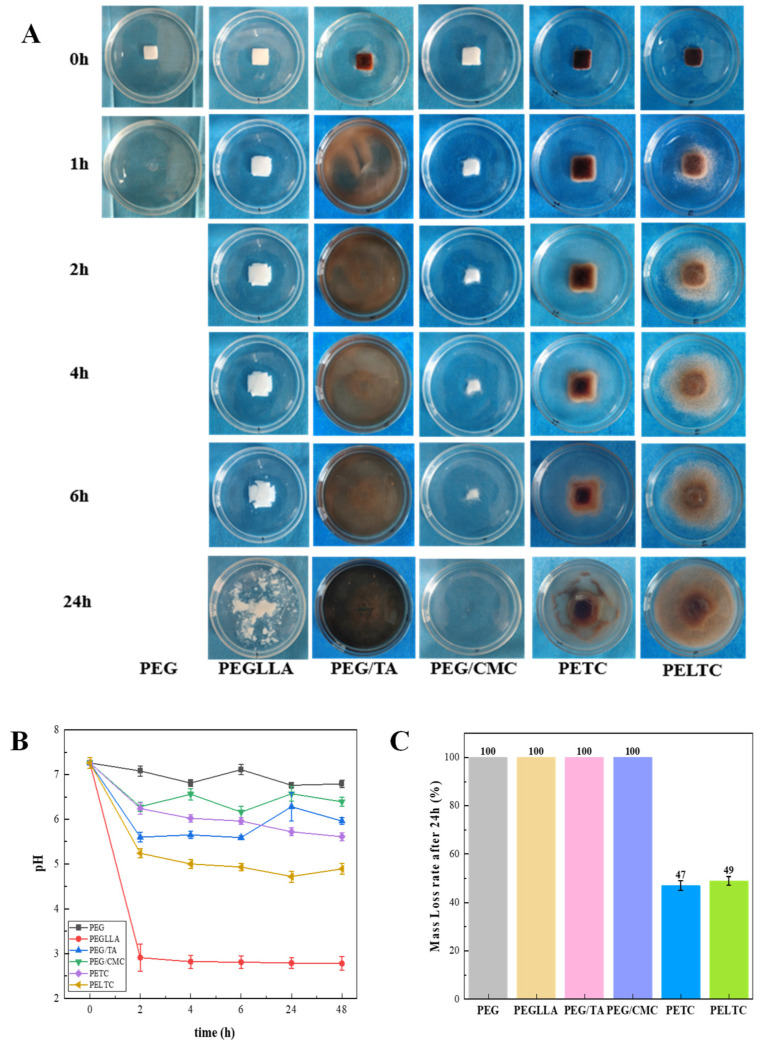
Solubility of absorbable bone wax in water: (**A**) The solubility of the sample in water. (**B**) The change in pH of the sample dissolved in water over time. (**C**) The mass loss rate of the sample dissolved in water after 24 h.

The excellent solubility of PEG gives it good absorbability, making it widely used in the medical field. As an absorbable bone wax, good absorbability is an essential characteristic, but too rapid absorption can cause the bone wax to lose its sealing effect, leading to rebleeding at the bleeding site and increasing surgical risk. Figure 3 shows the dissolution of absorbable bone wax in water. PEG samples demonstrated complete dissolution in water after one hour. Its mass loss rate after dissolving in water for 24 h is 100%. After PEG dissolves in water, the pH of the water is between 6.76 and 7.08. PEG is highly soluble in water because the oxygen atoms in its molecules have a higher partial charge (≈0.4–0.6 e) due to the induction effect, enabling them to form strong hydrogen bonds with water molecules. In addition, the “exclusion volume effect” of adjacent hydrophobic CH_2_ groups anchors the hydration layer, and the synergistic effect of these two factors gives PEG its excellent water solubility [54]. In contrast, PEGLLA samples exhibited undissolved particles within the sample and a loose external structure after six hours of dissolution in water. Following a 24 h dissolution period, the complete dissolution of the sample is observed, with only trace amounts of PLLA remaining as a hydrophobic phase dispersed in water. Following the filtration and drying processes, the sample exhibited a nearly complete disappearance, accompanied by a mass loss rate of 100%. After PEGLLA dissolved in water for 2 h, the pH dropped from 7.26 to 2.91. The system became acidic, and the pH fluctuated around 3.0. This is because PLLA contains hydrophobic groups, which slow down the dissolution rate [55], but PLLA has a low molecular weight, so it dissolves completely in water after 24 h. PLLA hydrolysis produces carboxyl groups, so the solution has an acidic pH, which accelerates the dissolution of PEGLLA in water [56]. PEG/TA demonstrated complete dissolution in water after one hour. Its mass loss rate after dissolving in water for 24 h is 100%. After PEG/TA dissolved in water for 2 h, the pH decreased from 7.26 to 5.60. After that, the pH is in the range of 5.59–6.28. This is because both PEG and TA contain hydroxyl groups, which form hydrogen bonds with water molecules, resulting in a faster dissolution rate. PEG/CMC can gradually dissolve in water, but its dissolution rate is slower than that of PEG and PEG/TA. After 6.0 h of dissolution in water, it still has not completely dissolved, but after 24 h, the sample is completely dissolved. Its mass loss rate after dissolving in water for 24 h is 100%. After PEG/CMC dissolved in water for 2 h, the pH decreased from 7.26 to 6.28. After that, the pH is in the range of 6.16~6.57. This is because a surface gel layer formed during the initial dissolution of CMC rapidly envelops the surrounding PEG, and the high viscosity of the gel layer significantly slows down the diffusion efficiency of PEG [57]. It has been demonstrated that both PETC and PELTC are partially dissolved in water for 24 h. The mass loss rates of PETC and PELTC after dissolving in water for 24 h are 47% and 49%, respectively. After PETC dissolved in water for 2 h, the pH decreased from 7.26 to 6.24. After that, the pH is in the range of 5.61~6.24. After PELTC dissolved in water for 2 h, the pH decreased from 7.26 to 5.24. After that, the pH is in the range of 4.72~5.24. This is because PEG, TA, and CMC form a porous network structure through hydrogen bonds. This network structure envelops PEG, preventing water molecules from entering the polymer interior. Furthermore, the strong hydrogen bonds between TA and CMC also restrict the expansion of CMC molecular chains, thereby slowing the dissolution rate. Due to the presence of PLLA in PELTC, the degradation product of PLLA is lactic acid, and the system is acidic. Therefore, the dissolution rate is slightly faster than that of PETC, and the mass loss rate is slightly higher than that of PETC. PETC and PELTC have slower dissolution rates and can maintain good sealing effects at bleeding sites, meeting the hemostatic requirements of absorbable bone wax. Thus, PETC and PELTC are suitable for use as absorbable bone wax.

### 2.4. Adhesion

**Figure 4:**
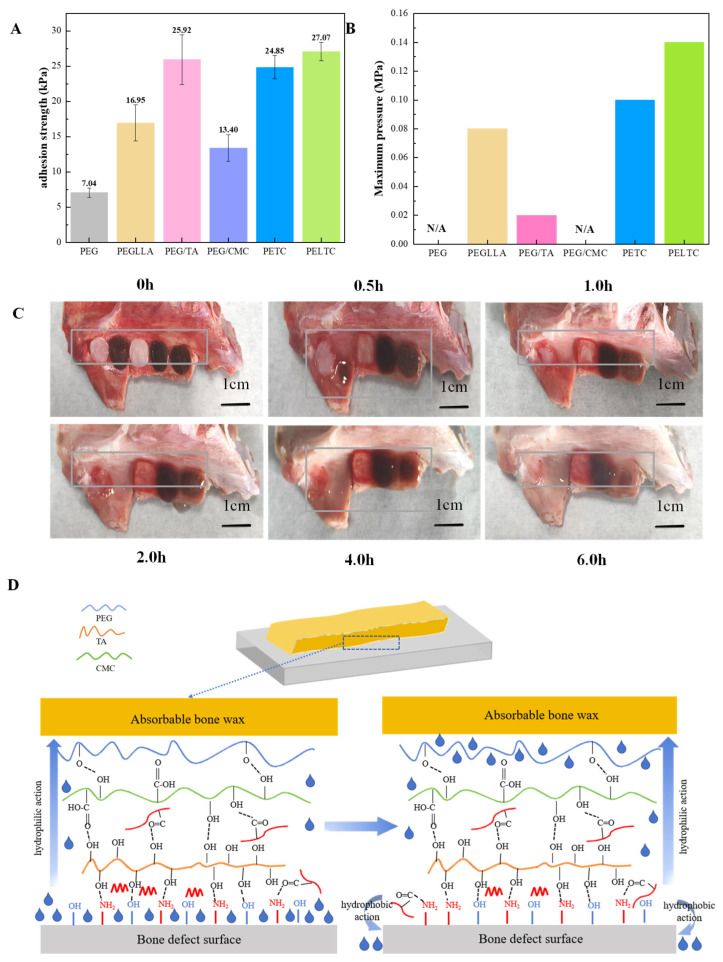
(**A**) Adhesion of absorbable bone wax to bovine bone. (**B**) Sealing properties of absorbable bone wax on catheters immersed in water. (**C**) Adhesion test of absorbable bone wax to moist pig bones (samples in the yellow box from left to right are PEGLLA, PEG/TA, PEG/CMC, PETC, and PELTC). (**D**) Adhesion mechanism of absorbable bone wax on moist bone surfaces. (Each sample is tested three times in the adhesion test, *n* = 3).

Good adhesion prevents bone wax from being washed away by blood or when cleaning wounds, thereby losing its hemostatic effect. In this paper, to increase the adhesion performance of bone wax, TA with polyphenol groups was introduced into the molecular system. As demonstrated in Figure 4A, PEG exhibits poor adhesion strength, and it is only 7.04 kPa. After adding 10% wt PLLA, the adhesion strength increased, and the adhesion strength of PEGLLA is 16.95 kPa. After adding 20% wt TA, the adhesive strength of the mixture increased significantly, with the adhesive strength of PEG/TA reaching 25.92 kPa. The addition of CMC also helps to increase the adhesion strength of PEG, but the improvement effect is limited, with the adhesion strength of PEG/CMC only reaching 13.40 kPa. PETC and PELTC have relatively superior adhesive strength, at 24.85 kPa and 27.07 kPa, respectively. Since PETC and PELTC have good adhesive strength and remain insoluble in water for 24 h, they were selected as the absorbable bone wax.

Absorbable bone wax has a good sealing effect on bleeding points in bone defects. The sealing effect of absorbable bone wax is simulated by applying pressure to a soaked tube in water. Normal human resting systolic blood pressure is ~120 mmHg (physiological baseline for tissue perfusion). However, hypertensive individuals show variability (e.g., 140–180 mmHg in untreated cases), and bleeding sites may transiently exceed baseline due to vasoconstriction or pulsatile flow, requiring strict sealing performance. In aqueous occlusion tests, catheters with occlusion material must tolerate > 200 mmHg to meet clinical needs—this threshold addresses extreme pathological blood pressure and reliable hemostasis under variable hemodynamics. As demonstrated in Figure 4B, PEG is highly soluble in water and exhibits poor adhesion properties, resulting in its inability to adhere to water-filled catheters and a lack of sealing capability. In contrast, due to the addition of hydrophobic PLLA, PEGLLA had a good sealing effect, allowing the catheter in water to maintain a pressure of 0.08 MPa (approximately 600 mmHg). Although PEG/TA improved adhesion due to the addition of TA, its sealing performance was generally poor due to its rapid dissolution rate in water. It can only withstand a pressure of 0.02 MPa (approximately 150 mmHg). PEG/CMC does not have a sealing effect on catheters. The PETC and PELTC models had been shown to demonstrate pressures of 0.10 MPa (approximately 750 mmHg) and 0.14 MPa (approximately 1050 mmHg), respectively, indicating their capacity for optimal sealing performance. Therefore, it is appropriate to choose it as absorbable bone wax.

In the context of bone wax utilization to seal bone defects, it is imperative to address persistent bleeding at the wound site. Therefore, the bone wax must maintain effective adhesion to the bone surface in a moist environment to ensure hemostatic efficacy. In this study, we simulated the application of absorbable bone wax at bone bleeding sites, with the results illustrated in Figure 4C. Our findings indicate that PEGLLA maintained adhesion to the bone surface after 1.0 h of immersion in water but detached after 2.0 h. In contrast, PEG/TA maintained adhesion within 0.5 h of immersion in water and detached at 1.0 h. PEG/CMC maintained adhesion within 1.0 h of immersion in water and detached at 2.0 h. Notably, both PETC and PELTC exhibited adhesion to the bone surface even after an extended soaking period of 6 h in water. PETC and PELTC have excellent adhesion to bone surfaces in water and can be used as absorbable bone wax.

Both PETC and PELTC exhibit excellent adhesion to bone tissue in water and can maintain their integrity for an extended period without disintegration. Among these, PELTC demonstrates superior adhesion and sealing properties due to the presence of PLLA, making it the preferred choice as an absorbable bone wax. The possible adhesion mechanism of PELTC in a humid environment is shown in Figure 4D:

TA is the primary adhesive agent due to its molecular structure, which is rich in phenolic hydroxyl groups (-OH). These groups can replace water molecules in the hydration layer on the bone surface and directly form stable hydrogen bond networks with the hydroxyl groups (-OH) of hydroxyapatite (HAP) and the amino groups (-NH_2_) of collagen [58]. The molecular mechanism of action is as follows: Tannic acid molecules rapidly recognize and adsorb onto collagen fibers or hydroxyapatite crystal surfaces at bone defects through hydrophobic interactions and hydrogen bonding. Tannic acid releases active hydroxyl groups, which undergo coordination reactions with calcium ions in the bone matrix. For example, it forms stable chelates with calcium ions [59]. As more tannic acid molecules deposit and oxidize, a dense metal-polyphenol network forms [60].

The hydroxyl (-OH) and carboxyl (-COO^−^) groups in CMC form hydrogen bonds with the phenolic hydroxyl groups of TA and the ether (-O-) bonds of PEG [61,62]. These bonds cause the various components to intertwine and form a stable, three-dimensional network structure [63]. This structure resists disintegration in water and maintains adhesion to tissue over time.

PEG has been demonstrated to enhance adhesion stability through interface optimization and stress buffering [64]. The material exhibits hydrophilic properties, rapidly absorbing moisture or blood from the tissue surface. This process disrupts the interfacial hydration layer, thereby eliminating the contact barrier between the material and the bone matrix [65]. Consequently, polymer chains can form more effective physical entanglement and chemical interactions with the moist tissue. In addition, the long-chain flexibility of PEG can buffer shear stress generated by physiological activities, such as joint movement. This adjustment of the distribution of interfacial forces through molecular chain flexion prevents adhesion failure caused by local stress concentration.

PLLA has been demonstrated to enhance adhesion through a combination of hydrophobic synergy and dynamic repair mechanisms. The hydrophobic segments of the polymer form secondary bonds with the hydrophobic regions of collagen, while the ester groups (-COO-) and the phenolic hydroxyl groups of the TA form hydrogen bonds to create “molecular bridges,” tightly connecting PLLA to the bone surface. The dynamic flexibility of low-molecular-weight PLLA enables rapid reconstruction of interactions through conformational adjustments of flexible segments when local hydrogen bonds are broken due to external stress. This process achieves dynamic repair and maintains long-term stability.

In addition, the strong and stable adhesion of absorbable bone wax underwater also depends on its unique molecular structure. The phenolic hydroxyl groups of TA, the carboxyl groups of CMC, and the ether bonds of PEG form a dynamic crosslinked network via hydrogen bonds. This network has been observed to restrict component movement, thereby reducing dissolution in water and dissipating external stresses, such as water flow impact through viscous flow [65]. The ester groups of PLLA have been found to further reinforce this physical crosslinking network. Furthermore, the hydrophilic continuous phase, which is composed of CMC and PEG, and the hydrophobic dispersed phase, which is composed of PLLA and TA, form a dual continuous network through mechanisms of water-induced phase separation. This structure integrates the wettability of the hydrophilic network with the mechanical strength of the hydrophobic microdomains, thereby markedly enhancing the material’s resistance to dissolution and adhesive strength.

### 2.5. Cytotoxicity and Hemocompatibility

As a material implanted into the human body, absorbable bone wax must possess excellent cellular compatibility and hemocompatibility. Although PEG, TA, CMC, and polylactic acid have been demonstrated to be non-cytotoxic, biodegradable, and possess good biocompatibility, further validation is required before practical application. Assessing material cytotoxicity by directly contacting a leaching solution of the bone wax.

**Figure 5:**
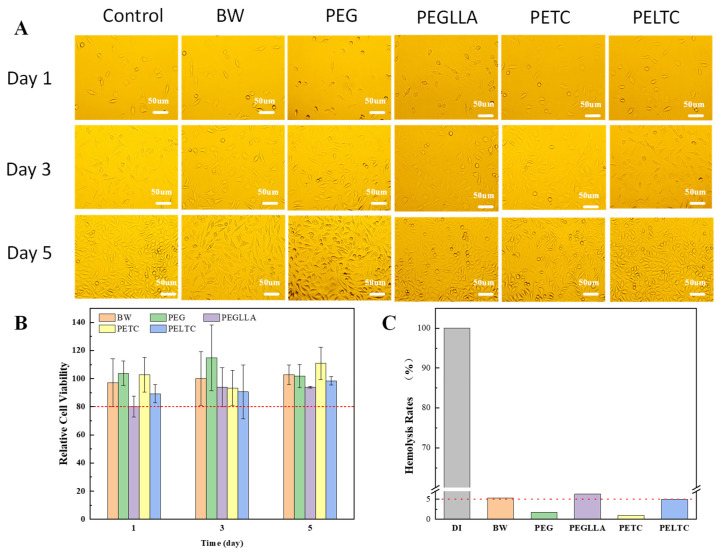
Cytotoxicity and hemocompatibility of absorbable bone wax: (**A**) Optical pictures of L929 cells cultured with a leaching solution of absorbable bone wax for 1, 3 and 5 days. (**B**) relative cell viability. (**C**) Hemolysis rate of absorbable bone wax. (The red dashed represents the qualified standard for cell compatibility and blood compatibility.)

Most cells exhibited a spindle shape, and cell numbers increased significantly. By day 5, cell numbers surged dramatically, completely filling the field of vision. Compared to the control group, cell densities in the leachates of several samples remained largely comparable to the control at each time point (Figure 5A). As shown in Figure 5B, the cell viability of all samples remained above 80%, with BW, PETC, and PELTC exhibiting viability rates exceeding 85%. This indicates that the prepared absorbable bone wax PELTC possesses excellent biocompatibility.

Blood compatibility testing has confirmed the biocompatibility of absorbable bone wax. Generally, a low hemolysis rate (<5%) of red blood cells (RBCs) is considered an indicator of qualified blood compatibility for biomaterials [66]. PEGLLA exhibited the highest hemolysis rate at 6.32%, indicating that PLLA has relatively poor blood compatibility. The hemolysis rates of PEG, PETC, and PELTC were 1.74%, 1.01%, and 4.95%, respectively, demonstrating excellent blood compatibility (Figure 5C). Therefore, the blood compatibility of both absorbable bone waxes, PETC and PELTC, meets the required standards.

### 2.6. Assessment of Hemostasis Performance

**Figure 6:**
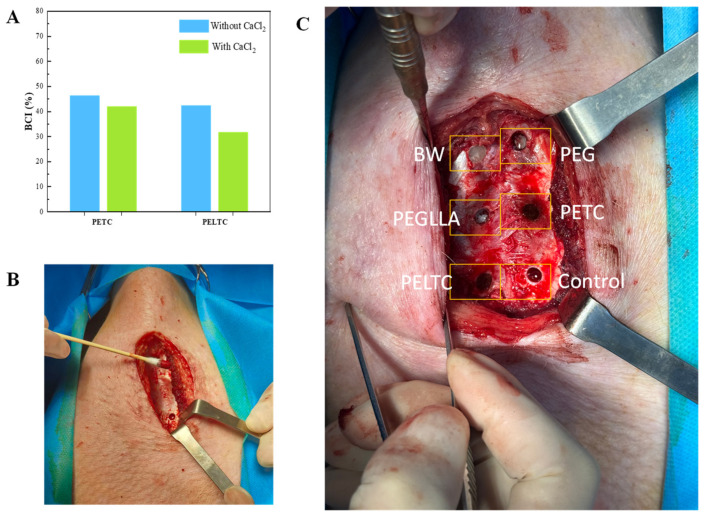
Hemostatic performance of different bone waxes: (**A**) Blood-clotting index (BCI). (**B**) Illustration of bleeding at the bone defect site, (**C**) Stop bleeding at the bleeding point.

The hemostatic performance of absorbable bone wax was evaluated using the blood-clotting index (BCI) assay. Absorbable bone wax was incubated with the same amount of citrated whole blood (extracted from a healthy pig) or recalcified whole blood (citrated whole blood mixed with 10% 0.2 M CaCl_2_ solution) to measure BCI (without CaCl_2_) or BCI (with CaCl_2_), respectively. As shown in Figure 6A, without the addition of CaCl_2_, the BCI values for PETC and PELTC were 46.39% and 42.41%, respectively. After adding PELTC, the BCI values for PETC and PELTC decreased to 42.03% and 31.80%, respectively. According to reports, the BCI values for medical gauze and commercial starch-based absorbable hemostatic agents (Arista) exceed 80% and 60%, respectively [67]. Therefore, the BCI values of PETC and PELTC are significantly lower than those of gelatin sponge and Arista. Lower BCI indicates faster clotting, demonstrating superior pro-coagulant activity. This is because the TA in PETC and PELTC contains a large number of phenolic hydroxyl groups. The phenolic hydroxyl groups in TA are easily oxidized into quinones. These quinones can mimic the oxidative microenvironment at sites of vascular injury, activating coagulation factor XII while simultaneously promoting platelet activation and aggregation. Therefore, PETC and PELTC not only maintain excellent adhesion and sealing properties at bone defects in moist environments but also exhibit superior blood compatibility and coagulation activity, making them an ideal choice as an absorbable bone wax.

Different types of bone wax were used to stop bleeding at the bone defect site of the pig sternum. The hemostatic effect was evaluated by testing the amount of bleeding and bleeding time. Table 1 and Figure 6C evaluate the hemostatic performance after bone wax implantation. In the absence of bone wax implantation, bleeding persisted at the bone defect site, with a bleeding time exceeding 60 min and a blood loss of 2139 mg. Following PEG implantation, the bleeding time and blood loss decreased rapidly to 2.15 min and 36.5 mg, respectively. Following the implementation of PEGLLA, the observed bleeding time and blood loss were 1.85 min and 34.1 mg, respectively. Given the relatively rapid dissolution rates and poor sealing performance of PEG and PEGLLA within the body, these materials nevertheless induced minor bleeding. The implantation of BW, PETC, and PELTC into the bone defect site resulted in immediate hemostasis. Hemostatic efficacy was superior to that of CHI-C/DACNC hydrogel (bleeding volume: 0.9 ± 0.3 g/10 min) [23] and CaPBW (bleeding volume: 0.074 ± 0.120 g) [68]. This is mainly due to the excellent adhesion and sealing properties of PETC and PELTC in moist environments. Not only can they act as a physical barrier to seal bleeding sites in bone defects, but the PEG they contain can also absorb blood, further enhancing the hemostatic effect. Therefore, absorbable bone wax PELTC has excellent hemostatic properties for bone defects.

### 2.7. In Vivo Degradation of Absorbable Bone Wax

**Figure 7:**
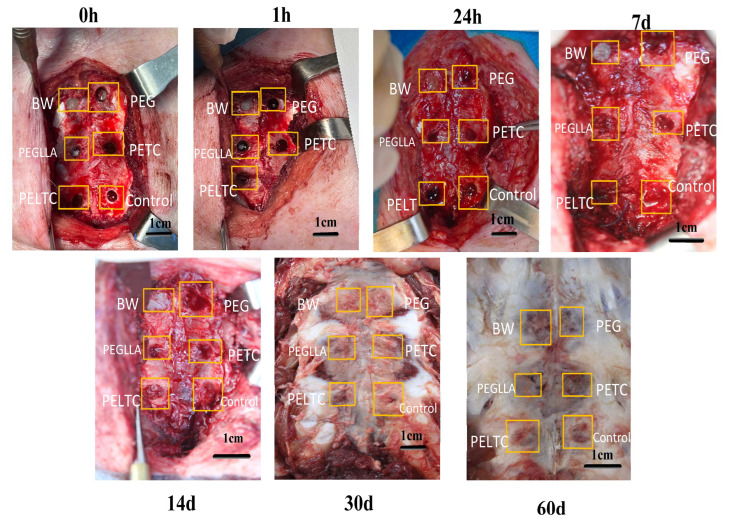
Degradation of the implanted sample in the porcine sternum.

In order to ensure the efficacy of hemostasis, bone wax must possess immediate hemostatic capabilities. Furthermore, absorbable bone wax must preserve partial structural integrity before new tissue growth to maintain hemostatic efficacy, promote wound healing, and prevent infection. And it needs to degrade within a specific time frame so it does not mess with bone healing. As illustrated in Figure 7, the dissolution of absorbable bone wax samples following implantation in swine subjects was investigated. Following the implantation of the PEG into the sternum of the pig, complete dissolution occurred within one hour, accompanied by bleeding at the bone defect site. In contrast, following the implantation of the PEGLLA into the pig’s bone, partial dissolution occurred after one hour, accompanied by minor bleeding. Following 24 h, both PEG and PEGLA had undergone complete dissolution. Both posed risks of secondary bleeding and infection at the bone defect site. Therefore, PEG and PEGLLA are not suitable for use as absorbable bone wax. Following a one-hour implantation period into porcine bone, BW, as well as PETC and PELTC, exhibited no signs of dissolution or bleeding. Furthermore, following 24 h, PETC and PELTC had not yet undergone complete degradation.

7 days postoperatively, BW remained undegraded, while PEG and PEGLLA were completely degraded. PETC and PELTC showed partial degradation. Except for the BW defect, all other defect sites exhibited minor filling with newly formed tissue, indicating a healing trend. 14 days postoperatively, BW remained intact while PEG, PEGLLA, PETC, and PELTC were completely degraded. New tissue filled all defect sites except the BW defect, demonstrating a clear trend toward healing. It can thus be seen that the degradation cycles of PETC and PELTC are essentially aligned with the growth cycles of newly formed tissue. On day 7, new tissue had partially grown, and PETC and PELTC were partially retained, still providing some hemostatic effect. After day 14, new tissue had fully grown, and PELTC had completely degraded, avoiding foreign body reactions and not affecting bone healing. After 30 and 60 days, the bone defects in all samples were completely covered by newly formed tissue.

### 2.8. Bone Repair

**Figure 8:**
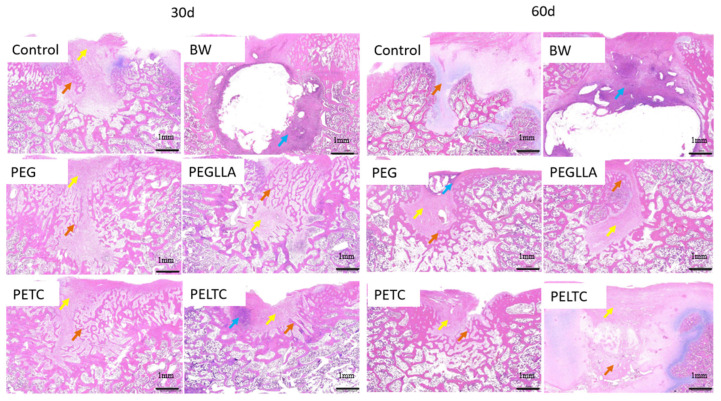
Histological evaluation of bone regeneration at 4 and 8 weeks following bone grafting. Red indicates newly formed bone tissue, yellow denotes fibrous tissue, and blue represents inflammatory cells. Scale bar: 400.0 µm.

This study employed a porcine sternal defect model to evaluate the bone regenerative capacity of absorbable bone wax. A cylindrical defect measuring 5.0 mm in diameter and 4.0 mm in thickness was created in the sternum. Subsequently, the defect site was treated with either non-absorbable bone wax (BW) or absorbable bone wax. Histological analysis of bone regeneration at 4 weeks and 8 weeks was performed using hematoxylin and eosin (H&E) staining (Figure 8). At 30 days and 60 days, the blank control group, BW group, and test groups (PEG, PEGLLA, PETC, and PELTC) showed localized damage to the sternal tissue. In the control and test groups, varying degrees of bone and fibrous tissue proliferation were observed in the damaged areas, accompanied by neovascularization. In contrast, the BW group exhibited fibrous encapsulation around the material-filled regions and significant inflammatory cell infiltration, with no bone proliferation. At 30 days, the fibrous tissue proliferation in the bone defect area of the control group was grade 3, and bone tissue proliferation was grade 1. The BW group had an inflammatory cell infiltration grade of 5, a fibrous tissue proliferation grade of 2; no bone tissue proliferation was observed, and the bone tissue repair effect was the poorest. In the experimental group, the new bone tissue proliferation grades in the defect areas of PEG, PEGLLA, and PETC were 2, 3, and 2, respectively, and the trabeculae of the new bone were slender in shape. The grade of bone tissue proliferation in the PELTC defect area is 1, the same as the control group, and the grade of inflammatory cell infiltration is 2. At 60 days, the control group showed grade 4 bone tissue proliferation in the sternal lesion area and grade 1 fibrous tissue proliferation, whereas in the BW group, inflammatory cell infiltration was grade 3, with no bone tissue proliferation observed. In the bone defect areas of PEG, PEGLLA, PETC, and PELTC, varying amounts of new bone tissue can be observed, accompanied by a large amount of fibrous tissue proliferation. Among them, the PETC group shows a bone tissue proliferation grade of 3, indicating slightly better bone repair, while the other materials all have a bone proliferation grade of 2. PETC and PELTC completely degrade after 30 days, without interfering with bone healing. Over time, bone tissue gradually increases at the defect site. In PELTC, degradation of PLLA leads to inflammation around the bone defect, which is not conducive to bone healing. Therefore, the bone repair effect of PELTC is slightly worse than that of PETC. PETC has the best bone repair effect due to the antibacterial properties of TA and the fact that its degradation products do not cause inflammatory reactions.

## 3. Materials and Methods

### 3.1. Materials

Polyethylene glycol (PEG), Mn = 2000 and Mn = 400, from Shanghai Macklin Biochemical Co., Ltd. (Shanghai, China). Heat-stable refined L-lactic acid (90%) from Wuhan Sanjiang Space Good Biotech Co., Ltd. (Guangshui, China), and Sn(II) octoate from Sinopharm Chemical Reagent Co., Ltd. (Shanghai, China). Tannic acid (AR) was obtained from Chengdu Huaxia Chemical Reagent Co., Ltd. (Chengdu, China). Carboxymethyl cellulose sodium (LR) was purchased from Chengdu Kelong Chemical Co., Ltd. (Qionglai, China). Non-absorbable bone wax (BW) was purchased from Sanhe Sanyou Medical Equipment Factory (Shanghai, China).

Electronic balance: LT502, Changshu Tianliang Instrument Co., Ltd. (Changshu, China). Dual Digital Display Constant Temperature Magnetic Stirrer, HJ-4B, Changzhou Surui Instrument Co., Ltd. (Changzhou, China). Heat-Collecting Constant Temperature Magnetic Stirrer, DF-101S, Gongyi Yuhua Instrument Co., Ltd. (Gongyi, China).

### 3.2. Preparation of Absorbable Bone Wax

**Figure 9:**
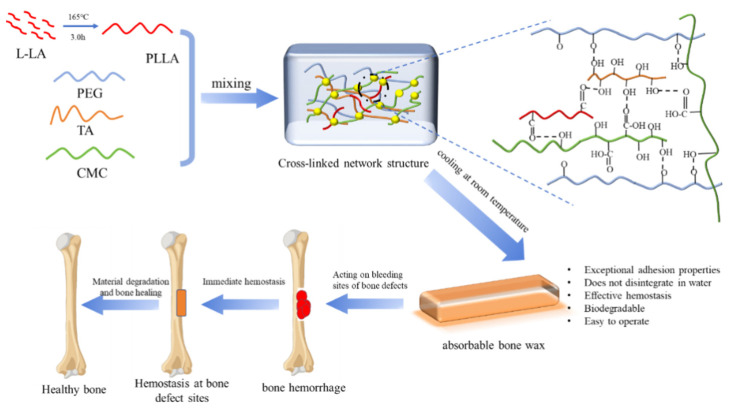
Preparation process of absorbable bone wax.

The preparation process of absorbable bone wax is shown in Figure 9. At 165 °C, a specific quantity of L-LA was weighed and placed in a three-neck flask to be preheated. After purging with nitrogen for 30 min, 0.8% of stannous acetate was added as a catalyst, relative to the mass of the L-LA. The reaction was carried out under magnetic stirring for 3 h, yielding a low-molecular-weight polymer (PLLA). According to Table 2, each component was added to a beaker. Subsequently, the beaker was placed on a magnetic stirrer and stirred to react for 0.5 h under the conditions of 80 °C and 600 rpm. After the reaction was completed, the beaker was removed and cooled to room temperature, yielding the absorbable bone wax.

### 3.3. Plasticity

Place the sample in the palm of your hand. Clasp both palms together for 10–20 s, then use your fingertips to knead, press, and stretch it. Observe whether it can be quickly shaped into a ball, thin sheet, or strip without crumbling or separating into layers.

### 3.4. Dissolution Performance Test

1.0 g of each sample was weighed and placed in a Petri dish containing purified water, and their dissolution behavior in water was observed. After 24 h, the residual samples were removed, rinsed with purified water, vacuum-dried, and then weighed. The mass loss rate was calculated, and the pH of the soaking solution for each sample was measured.

### 3.5. Adhesion Strength Test

Polished bovine bone slices (28 cm × 1.5 cm × 0.6 cm) were fixed to the clamping ends at both sides of the testing machine. Absorbable bone wax was applied to one end of a bone slice, and then another bone slice was manually pressed against it for 3–5 s to achieve bonding, with a bonding area of 10 mm × 15 mm. Peel tests were immediately conducted at a speed of 50 mm/min. The maximum load (N) at failure was recorded as the adhesive force, and the adhesive strength was calculated by dividing the maximum load by the bonding area. Each group of samples was tested in parallel three times, and the results were averaged.

### 3.6. Simulated Adhesion Experiment at Bleeding Sites

Samples were separately adhered to the surfaces of fresh porcine bones. Subsequently, the porcine bones together with the samples were immersed in water, removed at regular intervals, and the adhesion status of the samples on the bone surfaces was observed.

### 3.7. Sealing Performance Test

One end of a hose was completely sealed with absorbable bone wax and placed in water. Nitrogen gas was slowly introduced through the other end of the hose, and the air leakage of the hose was observed. The maximum nitrogen pressure under the airtight condition was recorded.

### 3.8. Fourier Transform Infrared Spectroscopy (FTIR) Analysis

An appropriate amount of powder samples was taken and prepared using the KBr pellet method. The molecular structure was determined using a SHIMADZU IRTracer-100 Fourier transform infrared spectrometer (Shimadzu Corporation, Kyoto, Japan). The test conditions were as follows: wavenumber range of 400–4000 cm^−1^, 16 scans, resolution of 4 cm^−1^, KBr detector, and infrared light source.

### 3.9. Differential Scanning Calorimetry (DSC) Analysis

The crystallization behavior of the materials was determined using a NETZSCH DSC 214 Polyma differential scanning calorimeter (NETZSCH-Gerätebau GmbH, Selb, Germany) under a nitrogen-protected atmosphere. Before the test, samples (7–8 mg) were weighed, placed in crucibles, and then put into the calorimeter. The samples were heated from room temperature to 100 °C at a rate of 10 °C/min, and their thermodynamic behavior was recorded.

### 3.10. Scanning Electron Microscopy (SEM) Characterization

Samples were first frozen in a refrigerator, then transferred to a freeze dryer for lyophilization for 8 h, and subsequently frozen in liquid nitrogen for 12 h. After being brittle-fractured, the cross-sectional morphology of the composites was observed. For surface observation, the samples were subjected to vacuum gold-sputtering treatment, and characterization was performed using a ZEISS Sigma 360 scanning electron microscope (Carl Zeiss AG, Oberkochen, Germany) with an accelerating voltage of 5 kV.

### 3.11. Rotational Rheological Tests

The rheological properties of the absorbable bone wax were determined using a NETZSCH Kinexus Prime lab+ rheometer (NETZSCH-Gerätebau GmbH, Selb, Germany). The test conditions were as follows: temperature of 37.0 °C, 40.0 mm parallel plate, and plate spacing of 1.0 mm. Frequency sweep tests were conducted at a strain level of 1.0% with an oscillation frequency range of 0.1–100.0 rad·s^−1^, and the storage modulus (G’) and loss modulus (G”) were recorded.

### 3.12. Cytotoxicity Experiments

The cytotoxicity of the bone wax was evaluated via the CCK-8 (Cell Counting Kit 8, Bisharp, Hefei, China) assay using L929 cells (mouse fibroblast cells) directly contacting a leaching solution of the bone wax. Mouse fibroblast cell line L929 was purchased from ABM Inc. (Richmond, BC, Canada; Catalog No. T1904). The leaching solution was prepared by soaking the sterilized bone wax in DMEM (Gibco, Shanghai, China) at a concentration of 100 mg/mL for 24 h at 37 °C, and then the supernatant was collected by 0.45 μm filter tip filtration, followed by dilution to 10 mg/mL and supplementing with 10% (*v*/*v*) fetal bovine serum (FBS, Sijiqing, Hangzhou, China). 100 uL of L929 cell suspension (1 × 10^4^ cells/mL) was inoculated in a 96-well plate. After incubation in 5% CO_2_, at 37 °C for 24 h, the original cell culture medium was replaced with the leaching solution. At the preset time of 1, 3, and 5 days, CCK-8 was added to each well and subsequently cultured in the dark at 37 °C for 2 h. Then, the absorbance of the medium was measured with a microplate reader at 450 nm. The relative cell viability was calculated as Absadhesive/Abscontrol × 100%, where Absadhesive and Abscontrol represent the absorbance of the adhesive and blank control groups, respectively. The cell cultured with original medium served as the blank control [69].

### 3.13. Hemolysis Rate

First, mix 1 mL of whole blood from a pig with 10 mL of PBS buffer. Centrifuge at 1500× *g* rpm for 15 min to remove the supernatant, then wash three times with PBS buffer. Resuspend the collected pellet in PBS buffer to prepare a red blood cell (RBC) suspension (volume concentration of 2%). Incubate the RBC suspension with samples at a 0.1 g/mL concentration of extractant, deionized water (positive control), and PBS buffer (negative control) at 37 °C for 3 h. Determine the hemolysis rate by measuring absorbance at 540 nm, with 5 replicate samples per group [67].

The hemolysis percentage was calculated using the equation:

$$\mathrm{Hemolysis}\;\mathrm{ratio}\;(\%)=\frac{A_{\hbar }-A_{p}}{A_{t}-A_{p}}\times 100\%$$

where *A_h_*, *A_p_*, and *A_t_* are the absorbance values of the supernatant fraction of the samples, negative (PBS), and positive control (Deionized water), respectively.

### 3.14. Whole Blood Clotting Test

100 µL of whole blood was dropped onto 50 mg of sample in a plastic centrifuge tube. Each group was immediately added with CaCl_2_ aqueous solution (1 µL, 0.2 M) and incubated at 37 °C for 5 min. Thereafter, 2 mL of DI water was carefully added to the centrifuge tube to break/dissolve the unclotted RBCs, which were further incubated at 37 °C for 10 min. Finally, the different suspensions were centrifuged (1500× *g* rpm, 5 min) and the supernatant liquid was collected. The hemoglobin (HGB) content of each group was determined by the subsequent measurement of absorbance at 540 nm (Abssample). The HGB content of citrated whole blood (10 µL in 2 mL DI water) was measured as the reference (AbsBlank). Five duplicate samples were performed for each group. The blood-clotting index (BCI) of the sample in recalcified whole blood was calculated from the following equation: BCI (with CaCl_2_) index = Abssample/Absblank × 100%. Meanwhile, the BCI (without CaCl_2_) index of sample in citrated whole blood was also calculated, using the same procedures without addition of CaCl_2_ aqueous solution [70].

### 3.15. In Vivo Hemostatic Performance and Dissolution Assessment

The design of animal experiments and the handling of animals were reviewed and approved by the Laboratory Animal Ethics Committee (IACUC) of Sichuan Lilaiseen Biotechnology Co., Ltd. (Chengdu, China), with the ethics record number LLSN-2025175. The experiments were conducted in accordance with GB/T 35823-2018 «Laboratory Animals—General Requirements for Animal Experiments». Male Large White pigs (Chengdu Dayuan Agriculture and Animal Husbandry Technology Co., Ltd., Chengdu, China) were used as research subjects. After 7 days of acclimatization (temperature 20–28 °C, humidity 40–70%), a paired control method was adopted, and 2 pigs without physiological abnormalities (40–50 kg, male, 3–4 months of age) were selected for the experiment. Establish an absorbable bone wax pig sternum defect model, selecting 6 areas on the sternum of each experimental pig to serve as the control group and the BW, PEG, PEGLLA, PETC, and PELTC bone wax groups, respectively. All animals were fasted (water allowed) for 12 h prior to surgery. Induction was achieved using a combination of Telazol and Xylazine, followed by maintenance with isoflurane. After anesthesia takes effect, the chest area is sterilized. The test pig is secured in a supine position on the operating table. The surgical site is disinfected and draped, then locally infiltrated with 1% lidocaine for anesthesia. A vertical incision of 4–5 cm in length was made at the midline of the sternum to expose the sternal surface. Six circular holes with a diameter of 5 mm and a depth of 4 mm were drilled into the sternum to reach the cancellous bone. Positions 1, 2, 3, 4, and 5 were filled with bone wax (BW), PEG, PEGLLA, PETC, and PELTC, respectively, with position 6 serving as a blank control. After surgery, the chest soft tissue and skin were sutured in sequence, ensuring no infection in the surgical area. Postoperatively, antibiotics were injected into the muscles for 1–3 days to prevent infection, once per day, and all experimental animals were strictly housed individually. After the implantation of the material, measure the hemostasis time and blood loss, observe the hemostatic effect at 1 h and 24 h post-operation, and monitor the degradation of the test material at 7, 14, 30, and 60 days post-operation.

### 3.16. Bone Healing Status

One experimental pig was selected for euthanasia at 30 days and 60 days for gross anatomy and histopathological diagnosis. After the sternum of the test pigs was fixed, decalcification, paraffin embedding and sectioning were carried out, and HE staining was performed. Under the microscope, observe the formation of trabeculae, the development of fibrous capsules, the repair of bone tissue, etc., and perform a four-grade scoring. The corresponding descriptions of relative lesion severity and scoring are shown in Table 3.

## 4. Conclusions

In summary, we successfully prepared PEG-based absorbable bone wax by combining PEG, TA, CMC, and low-molecular-weight PLLA to construct a crosslinked network structure using hydrogen bonds. This bone wax shows good adhesion properties when applied to bone tissue surfaces, exhibiting stable adhesion even in aqueous environments. Experiments involving animal models have demonstrated the hemostatic efficacy of the substance under investigation to be comparable to that of traditional bone waxes. Consequently, this novel absorbable bone wax is indicated for achieving instant and stable hemostasis in complex bone defects and areas with significant bleeding. Its hemostatic mechanism, in addition to its physical barrier effects, involves the absorption of blood and adhesive sealing of the bleeding point, which further accelerates the hemostatic process. Furthermore, the degradation of this bone wax occurs within a predetermined timeframe, thereby averting the onset of a foreign body reaction while ensuring unobstructed bone healing. While PELTC achieved immediate hemostasis comparable to traditional wax and degraded within 14 days, longer-term bone healing (60 days) was suboptimal, potentially due to acidic degradation products. Further optimization of the molecular weight and content of polylactic acid (PLLA) or the addition of buffering agents may be required.

## Figures and Tables

**Table 1:** Hemostasis of different bone waxes.

Sample	Clotting Time (min)	Bleeding Weight (mg)
Control	Over 60	2139
BW	Immediate hemostasis	0
PEG	2.15	36.5
PEGLLA	1.85	34.1
PETC	Immediate hemostasis	0
PELTC	Immediate hemostasis	0

**Table 2:** Composition of absorbable bone wax.

Sample	PEG2000/ wt%	PEG400/ wt%	PLLA/ wt%	TA/ wt%	CMC/wt%
PEG	80	20	/	/	/
PEGLLA	90	/	10	/	/
PEG/TA	60	20	/	20	/
PEG/CMC	60	20	/	/	20
PETC	40	20	/	20	20
PELTC	30	20	10	20	20

**Table 3:** Description and Scoring of Relative Lesion Severity.

Grade	Degree of Lesion	Definition of Classification
0	Normal range	Under experimental conditions, considering factors such as age, sex, and strain, changes may occur, but under other circumstances, they might be considered deviations within the normal range.
1	Slight	The changes that occur are almost no more than the normal range of variations (i.e., minimal changes)
2	Mild	Lesions are easy to identify, but the severity is limited; the lesions may not cause any functional impairment; the affected tissue accounts for 11–20% of the examined tissue.
3	Moderate	The lesion is prominent and is likely to progress toward severity. It may cause limited dysfunction of tissues or organs; 21% to 40% of tissue is affected.
4	Severe	The lesions are severe and have developed into complete lesions, which are expected to cause significant tissue or organ dysfunction; the lesions involve 41–100% of the examined tissue area.

## Data Availability

The data presented in this study are available on request from the corresponding author due to privacy and confidentiality restrictions.

## Supplementary Materials

The following supporting information can be downloaded at https://www.mdpi.com/article/10.3390/ijms27010276/s1.
